# The Trauma PORTAL—A Blended e-Health Intervention for Survivors of Childhood Interpersonal Trauma: An Open-Label Pilot Study

**DOI:** 10.1089/tmr.2024.0020

**Published:** 2024-07-12

**Authors:** Tina Behdinan, Annie K. Truuvert, Aishat Adekunte, Nancy McCallum, Simone N. Vigod, Aysha Butt, David Rojas, Sophie Soklaridis, Dana C. Ross

**Affiliations:** ^1^ Department of Psychiatry, Temerty Faculty of Medicine, University of Toronto, Toronto, Canada.; ^2^ Women’s College Hospital and Research Institute, Toronto, Canada.; ^3^ The Wilson Centre, University of Toronto, Toronto, Canada.; ^4^ Centre for Addictions and Mental Health, Toronto, Canada.

**Keywords:** childhood interpersonal trauma, complex trauma, PTSD, psychoeducation, psychotherapy, virtual, evaluation

## Abstract

**Background:**

Adults with mental health symptoms stemming from childhood interpersonal trauma require specialized trauma-focused psychological interventions. Limitations in accessing treatment interventions for this population necessitate innovative solutions. This study explored the feasibility of a protocol for a blended e-health psychoeducational treatment intervention for this population called the Trauma PORTAL (Providing Online tRauma Therapy using an Asynchronous Learning platform), combining asynchronous online modules and weekly live virtual group sessions.

**Method:**

From October 2021 to February 2022, this prospective, single-arm study recruited participants who were waitlisted for trauma therapy at an academic hospital. The primary outcome was protocol feasibility, including recruitment, adoption, and intervention acceptability. Secondary outcomes were pre- and post-intervention post-traumatic stress disorder (PTSD) symptoms (PTSD Checklist for DSM-5 [PCL-5]), depression/anxiety/stress (Depression and Anxiety Stress Scale [DASS-21]), and emotion regulation (Difficulties in Emotion Regulation Scale [DERS-18]), which were compared using paired *t*-tests and presented as mean differences (MDs) and 95% confidence intervals (CIs).

**Results:**

A total of 66 participants (median age = 37, female = 61) were enrolled, and they completed on average 53.5% of the online modules. There were 51 (77%) participants who completed post-intervention questionnaires. Acceptability was very high, with 49 respondents (98%) reporting that the intervention increased their access to health care. There were reductions from pre- to post-intervention on the PCL-5 (49.1 vs. 36.7, MD −12.4, 95% CI 8.3–16.5), DERS-18 (51.8 vs. 48.8, MD −3.3, 95% CI 0.2–6.4), and DASS-21 (60.1 vs. 50.7, MD −9.4, 95% CI 2.3–16.6).

**Conclusion:**

The Trauma PORTAL intervention was feasible to implement, well-adopted, and highly acceptable in an ambulatory trauma therapy program. The findings show promising evidence for symptom reduction. Further evaluation of the Trauma PORTAL’s efficacy in a randomized trial is warranted.

## Introduction

Individuals who have experienced childhood interpersonal trauma (CIT), including physical, sexual, and emotional abuse or neglect, are at high risk of developing mental health conditions, including depression, anxiety, and post-traumatic stress disorder (PTSD) as adults.^1,2^ Few health care providers are adequately trained in evidence-based trauma-focused interventions for this population, resulting in long waitlists for care in many jurisdictions.^3,4^ Innovative solutions to improve care efficiencies are needed.

Psychoeducation is an important first step in the treatment of mental health conditions, including treating the sequelae of CIT.^5–8^ Psychoeducation goes beyond imparting information about how CIT influences the brain, behavior, and relationships; it also equips individuals with skills and strategies for emotional self-regulation.^9^ Studies, including a recent systematic review and meta-analysis, showed that phase-based interventions that incorporate a skill-based component are effective in treating people with complex trauma experiences, such as childhood sexual abuse.^10,11^ Foundational educational components may improve symptoms, even without additional interventions.^12,13^ Some individuals will require further trauma-focused psychological interventions, which align with stepped care models that begin with lower-intensity services such as psychoeducation and only “step up” into higher-intensity services when the lower-intensity services are insufficient.^14,15^ The advantages of psychoeducational interventions include reducing the need for extensive therapist training, facilitating implementation in low-resource settings, and increasing health care capacity by enabling nonspecialist providers to deliver treatment.^15,16^ Further research, including randomized controlled trials (RCTs), are needed to evaluate the efficacy of treatment interventions, including psychoeducational components, in people coping with symptoms related to CIT.^17–19^

Electronic (e)-health interventions are being used with increasing frequency to improve access, efficiency, and effectiveness of care for mental health conditions.^20–24^ e-Health can reduce mental health care disparities, as individuals with geographic limitations, challenging work schedules, and disabilities can more easily access treatment.^25,26^ Recent e-health interventions combine synchronous therapist interactions with asynchronous self-paced elements to improve health care efficiency.^27,28^ These blended interventions show promise in the treatment of mental health issues, including PTSD, however, the majority of studies on blended e-health interventions for trauma tend to evaluate cognitive behavioral therapy-based strategies and do not specifically focus on people with a history of childhood trauma.^2,29,30^ To our knowledge, blended psychoeducational e-health interventions for adults dealing with the mental health consequences related to CIT have not been previously evaluated.

Our blended e-health intervention, the Trauma PORTAL (Providing Online tRauma Therapy using an Asynchronous Learning platform), previously referred to as electronic Resourced & Resilient,^31^ offers psychoeducational content for adults with a history of CIT. The PORTAL’s multimedia online modules can be completed independently by participants over 8 weeks, with optional 60-minute weekly virtual group sessions facilitated by highly trained clinicians to review the course material. The primary goal of this study was to assess the feasibility, usability, and acceptability of the Trauma PORTAL protocol among patients referred to a tertiary care trauma therapy program (TTP) to inform modifications needed prior to a large-scale RCT.

## Methods

### Study design and setting

This open-label feasibility study evaluated an intervention offered to patients referred to the TTP, a specialized trauma-focused psychotherapy clinic, at Women’s College Hospital (WCH), a university hospital in Toronto, Ontario, Canada. This open-label study design allowed for both researchers and participants to know what intervention was being administered. There was no control group. This study was conducted in accordance with the principles of the Declaration of Helsinki and approved by the Research Ethics Board at WCH.

#### TTP program description

The TTP at WCH is a specialized clinical service within the Department of Psychiatry, catering to adult survivors of childhood trauma. Referrals, accepted from physicians and nurse practitioners, undergo screening for eligibility criteria including age over 18, history of childhood trauma, and absence of recent psychiatric hospitalizations. Following clinical assessment, patients deemed suitable for outpatient psychoeducational interventions participate in an 8-week Resourced and Resilient Group, integrating evidence-based approaches like mindfulness, cognitive, body-oriented, and relational strategies. Subsequently, patients may opt for more focused healing pathway groups, such as healing through the mind, body, arts, or relationships, each employing tailored therapeutic strategies. Those completing at least two groups, demonstrating stability and goal alignment, may progress to time-limited individual trauma-focused psychotherapy or relational group therapy. This progressive approach ensures comprehensive support for survivors in addressing trauma-related challenges and fostering recovery.

### Participants

Patients waiting for care were invited to participate in the study via a recruitment flyer mailed to their home addresses, followed 2 weeks later by a telephone call to see if they were interested in participating. Research staff explained the study, screened potential participants for eligibility, and conducted informed consent procedures. Participants then met virtually with a TTP therapist for a 30-minute clinical assessment to confirm their ability to safely participate in the intervention and provide instructions for the asynchronous and synchronous components of the intervention. Once enrolled, participants completed a telephone baseline assessment with a research staff member and submitted baseline self-report measures via Research Electronic Data Capture (REDCap).^32^ Follow-up measures were also collected by self-report via REDCap post-intervention.

### Intervention

The Trauma PORTAL program consisted of an asynchronous component (online modules) and a synchronous component (weekly virtual group session). There are eight online modules that participants work through independently throughout the 8-week intervention. Module material was adapted from the 8-week manualized trauma psychoeducation group offered in the TTP for over 15 years (see Supplementary Appendix S3 for module content). Modules include videos, podcasts, audio clips, animations, worksheets, and quizzes so that participants can self-evaluate the knowledge gained as they move through the program. Modules were evaluated using qualitative methods during the development phase of this study.^31^ Participants have their username and login to the system and can access all the eight online modules at any time of day and can work through them at their own pace. There is no personal health information stored within the online system. The synchronous component was an optional, weekly, 1-hour Zoom group session facilitated by two trauma therapists from the TTP, where participants could ask questions or for clarification about the online module material. The same two trauma therapists were scheduled to co-facilitate each week of the 8-week group to foster a therapeutic context where participants and therapists could become familiar with each other and build rapport. Patient participants were divided into 3 cohorts, comprising up to 25 participants per cohort, to limit the size of the weekly virtual groups. During these sessions, participants could interact with facilitators and other participants who attended. All participants were registered with the TTP, and the weekly group session attendance was charted as a patient visit in the hospital’s electronic medical record system.

### Inclusion/exclusion criteria

TTP patients who were on the waitlist for their first treatment group were invited to participate in the study and had to meet specific inclusion criteria. These included: (a) at least 18 years of age; (b) self-report of CIT (prior to age 18); (c) PTSD Checklist for DSM-5 (PCL-5) score ≥26; and (d) access to an appropriate device and internet connection to access the virtual intervention. Patients meeting one or more of the following criteria that suggested high clinical acuity were excluded: active substance use in the past 3 months, active symptoms of mania, psychosis, or suicidal ideation, and psychiatric hospitalization in the past 6 months. Participants were excluded if, based on a clinical assessment with a TTP therapist, there was a concern that they had significant difficulty with self-regulation and would instead benefit from a synchronous treatment in the TTP that offered more therapist support, or cognitive impairments that would impede understanding and processing of educational material, or significant case management needs that would result in lack of suitability for asynchronous online group therapy.

#### Baseline measures

At baseline, the Mini International Neuropsychiatric Interview (MINI) Module A was conducted to assess for current and lifetime psychiatric diagnoses and severity. Participants completed a self-report baseline questionnaire that inquired about sociodemographic information, concurrent health service utilization, and the extent of their childhood trauma experiences measured using the Adverse Childhood Experiences (ACE) scale.^33^ ACE scores range from 0 to 10, each point on the scale representing a specific type of traumatic childhood experience, thus, indicating the cumulative number of traumatic experiences a participant has experienced.^33^ Research indicates that a score of 4 or more indicates a significantly increased risk for negative health outcomes.^34^

### Primary outcome

The primary outcome of this study was the feasibility of the Trauma PORTAL protocol, including the feasibility of recruitment and retention for the research protocol, program adoption, usability, and acceptability. The recruitment rate was defined as the number of enrolled participants per month, and retention throughout the study as the proportion of participants completing follow-up measures. Adoption (module completion and drop-in session attendance) was measured from when participants received their login for the Trauma PORTAL until the 8-week intervention was completed. Acceptability and usability were measured with a post-intervention acceptability questionnaire, an optional iterative feedback form sent to participants weekly for comments on the online platform and module, and the estimated time spent on the modules and the reason for incomplete modules. The intervention acceptability questionnaire was adapted from a validated questionnaire measuring the acceptability of telehealth services. Attitudes towards the internet were measured using the General Internet Attitude Scale (Supplementary Appendix S2).^35^

### Secondary outcomes

PTSD symptoms were measured using the PCL-5,^36^ depression and anxiety symptoms using the Depression and Anxiety Stress Scale (DASS-21),^37^ and emotion regulation using the Difficulties in Emotion Regulation Scale (DERS-18).^38^ Research indicates that a PCL-5 score of 31–33 is a typical cut-off score for making a provisional diagnosis of PTSD, and a decrease in post-intervention scores by 10 points represents a clinically significant change.^39^ As a formal diagnosis of PTSD is not necessary to access services in the TTP, we utilized the subthreshold score for our inclusion criteria to capture individuals representative of our patient population more fully and to evaluate the cut-off score for a future RCT. The DASS-21 comprises three subscales, including depression, anxiety, and stress.^37^ In comparison, the DERS comprises six subscales that assess an individual’s awareness and acceptance of their emotions, along with their capacity to regulate their emotions and impulses effectively. There are no official clinical thresholds for the DERS-18. However, it is a well-validated and reliable scale used to assess changes in an individual’s ability to regulate emotion over time.^38^ All clinical measures were assessed at baseline and post-intervention using REDCap. As additional proxies for intervention effectiveness, we also conducted a post-intervention chart review identifying the next steps participants took in the trauma therapy treatment in the TTP, i.e., whether they needed additional trauma therapy after the PORTAL, and if so, whether the assessing health care provider felt that they were ready to progress to a psychological intervention (vs. requiring a repeat of the PORTAL material via a fully synchronous version of the psychoeducational intervention).

### Statistical analysis

To assess feasibility, we calculated the proportion of participants who were eligible once contacted, and the proportion who enrolled once screened as eligible. Retention was calculated as the proportion of enrolled participants who completed the post-intervention questionnaires. We calculated the proportion of enrolled participants who engaged in module completion and the drop-in sessions in order to assess program adoption. Acceptability and usability were assessed using participant feedback on the post-intervention acceptability questionnaire and a weekly, optional iterative feedback form. For each symptom scale measure, the pre- and post-intervention scores were compared using paired *t*-tests, with the generation of mean differences and 95% confidence intervals. We also calculated the proportion of participants who met a clinically significant threshold PCL-5 score of 31 before and after the intervention, and the proportion of participants whose PCL-5 scores changed by a clinically significant degree (≥10 points) from baseline.

## Results

### Recruitment and retention

**FIG. 1.**
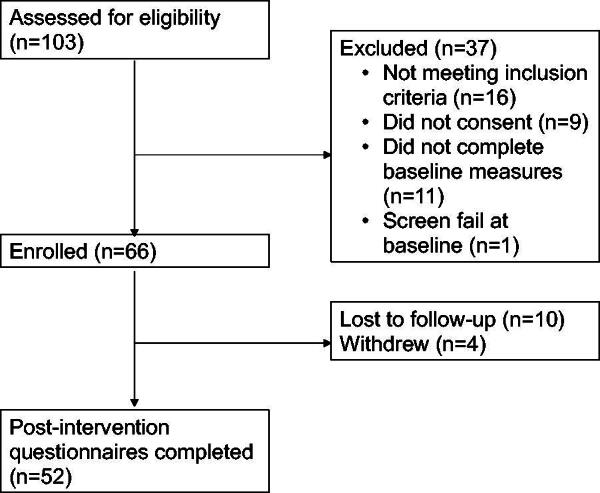
Flowchart recruitment and retention (*n* = 66).

From October 2021 to February 2022, 275 TTP patients were sent the study flyer via mail. Of these patients, 119 expressed interest to learn more about the study and were contacted by research staff, 16 were not interested in participating in the study, and 103 (86.6%) were screened for eligibility. We excluded those with PCL-5 <26 (*n* = 10) and those (*n* = 6) with an active substance use disorder, acute mania, active suicidality, recent psychiatric hospitalization, and no internet access. Of the 87 potentially eligible participants, 66 (75.9%) were enrolled in the study, representing an overall enrollment of 64.1% and a recruitment rate of 13.2 participants/month. Of the 66 enrolled participants, 52 (77.3%) completed the post-intervention questionnaires. Four participants withdrew from the study before completing the intervention, and the remaining 10 were lost to follow-up. Two participants were hospitalized during the study related to mental health concerns, and both chose to continue in the study. There were no other participants who reported any safety concerns and no serious adverse events. No participant dropped out of the study for safety reasons (Fig. 1).

About 39.4% reported an annual household income of less than CAD 40,000 (i.e., below the national median household income). The mean score on the ACE was 5.03 (standard deviation [SD] = 2.33) and 74.2% of the patients had a score of at least 4 (Table 1).

**Table 1.** Baseline Demographic and Clinical Characteristics of Study Participants (*n* = 66). Characteristics Are Shown as *n* (%) Unless Otherwise Specified

	Participants, *n* (%)
Median (IQR) age	37 (IQR 28–44)
Gender-identifying as women	61 (92.4)
Highest level of education	
University or college	48 (72.7)
Some university or college	15 (22.7)
Elementary/secondary	3 (4.5)
Employment status	
Full time	30 (45.5)
Part time	13 (19.7)
Unemployed	20 (30.3)
Prefer not to say	3 (4.5)
Annual household income ($)	
>80,000	17 (25.8)
40,000–80,000	10 (15.2)
20,000–40,000	18 (27.3)
<20,000	8 (12.1)
Prefer not to say	13 (19.7)
Ethnicity	
White/European	40 (60.6)
East Asian/Asian	11 (16.7)
Black/Afro-Caribbean	7 (10.6)
Latino/Hispanic	4 (6.1)
South Asian/Indian	3 (4.5)
Middle Easter/Arab	3 (4.5)
First Nations/Inuit/Metis	1 (1.5)
Prefer not to say	0 (0)
Don’t know	2 (3.0)
Other	3 (4.5)
Mean ACE score (SD)	5.03 (2.33)
Current psychiatric diagnosis (MINI)	
PTSD	36 (54.6)
Agoraphobia	5 (7.6)
Panic disorder	17 (25.8)
Major depressive episode	21 (31.8)
Generalized anxiety disorder	28 (42.4)
Obsessive compulsive disorder	10 (15.2)
Social anxiety disorder	21 (31.8)
Current alcohol use	
Yes	28 (42.4)
No	38 (57.6)
Current cannabis use	
Yes	29 (43.9)
No	36 (54.6)
No response	1 (1.5)
Cigarette smoking	
Never smoked	39 (59.1)
Past	19 (28.8)
Current	3 (3.5)
Concurrent psychiatric support(s)	
Psychiatrist	20 (30.3)
Psychologist	11 (16.7)
Social worker	16 (24.2)
Psychotherapist	8 (12.1)
Family physician	17 (25.8)
Other	4 (6.1)
None	12 (18.2)
Concurrent mental health treatment	
Yes	39 (59.1)
No	27 (40.9)
Current medical condition(s)	
Yes	47 (71.2)
No response	19 (28.8)
Currently taking medications, vitamins, supplements	
Yes	59 (80.3)
No	7 (19.7)

ACE, Adverse Childhood Experiences Questionnaire; IQR, interquartile range; MINI, Mini International Neuropsychiatric Interview; PTSD, post-traumatic stress disorder; SD, standard deviation.

### Adoption

Of the 66 participants, 58 (87.9%) engaged with the program in some way (i.e., attending groups, engaging with modules, or both) and 8 (12.1%) did not engage with any component (Table 2).

**Table 2.** Participant (*n* = 66) Engagement in Modules and Weekly Group Sessions

Module engagement	n (%)
0 modules	11 (16.7)
1–2 modules	11 (16.7)
3–4 modules	10 (15.2)
5–6 modules	8 (12.1)
7–8 modules	26 (39.4)

Weekly group sessions attended	n (%)
0 sessions	28 (42.4)
1–2 sessions	11 (16.7)
3–4 sessions	5 (7.6)
5–6 sessions	8 (12.1)
7–8 sessions	14 (21.1)

Five (7.6%) participants completed all modules and attended all optional drop-in sessions. Thirty-three completed at least 75% of the online modules, making them eligible to progress to other trauma groups in the TTP after the study.

### Acceptability and usability

**FIG. 2.**
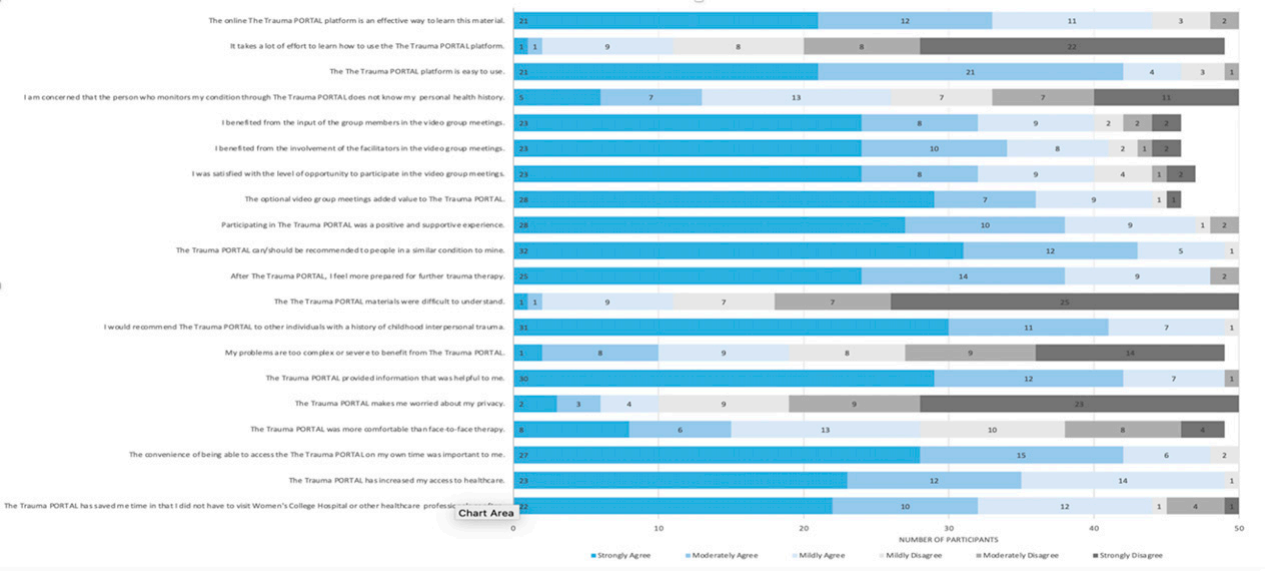
Intervention acceptability questionnaire showing participant ratings (*n* = 50).

Fifty participants completed the intervention acceptability questionnaire. 98% of the respondents agreed or strongly agreed that the Trauma PORTAL increased their access to health care, provided helpful information, and that they would recommend the Trauma PORTAL to others with a history of CIT (Fig. 2). 95.9% agreed that the convenience of accessing the Trauma PORTAL on their own time was important. Over 90% of the participants agreed that the Trauma PORTAL platform was easy to use.

Optional iterative feedback forms were completed by an average of 19.6 (SD = 5.4) participants each week. According to the responses, participants most often spent 30–60 min on modules, with a mean of 8.5 (43.3%, SD = 2.1) participants reporting this figure each week (see Supplementary Appendix S1). The most common reason for not completing a module was that participants needed more time that week.

### Clinical symptoms

There were statistically significant changes in clinical measures from pre- to post-intervention across all three symptom scales (Table 3). The proportion of participants with a PCL-5 threshold score of 31 or greater was 89.8% at baseline and 53.1% post-intervention; this association was significant, *X*^2^(1, *n* = 66) = 16.2, *p* < 0.001. There were 27 (55%) participants with a clinically significant decrease in PCL-5 score of at least 10 points.

**Table 3.** Clinical Symptom Scale Scores of Participants Who Completed the Trauma PORTAL Intervention, Presenting Paired *t*-Tests with Mean Differences and 95% Confidence Interval Comparing Post- with Pre-Intervention

	Baseline, mean (SD)	Post-treatment, mean (SD)	Paired *t* (*p*)	Mean difference (95% CI)
PCL-5 (*n* = 49)	49.1 (13.12)	36.7 (16.42)	6.03 (<0.01)	−12.4 (−16.5, −8.25)
DASS (*n* = 44)				
Overall	60.1 (25.2)	50.7 (25.3)	2.67 (0.01)	−9.45 (−16.6, −2.31)
Depression	21.3 (10.68)	18.2 (10.68)	2.21 (0.03)	−3.09 (−5.91, −0.27)
Anxiety	16.9 (9.75)	14.8 (9.29)	1.52 (0.14)	−2.09 (−4.87, 0.69)
Stress	22.0 (9.62)	17.7 (8.62)	3.00 (0.01)	−4.27 (−7.15, −1.40)
DERS (*n* = 46)				
Overall	51.8 (13.9)	48.5 (12.4)	2.16 (0.04)	−3.30 (−6.39, −0.22)
Awareness	7.83 (3.06)	7.98 (2.55)	−0.46 (0.65)	+0.15 (−0.52, 0.82)
Clarity	7.72 (3.12)	7.61 (3.40)	0.33 (0.74)	−0.11 (−0.76, 0.55)
Goals	11.6 (3.17)	11.0 (2.73)	1.62 (0.11)	−0.63 (−1.42, 0.15)
Impulse	6.65 (3.19)	5.91 (2.53)	1.63 (0.11)	−0.74 (−1.65, 0.17)
Nonacceptance	9.52 (3.71)	8.48 (3.33)	2.44 (0.02)	−1.04 (−1.90, −0.18)
Strategies	8.5 (3.66)	7.57 (3.05)	1.88 (0.07)	−0.93 (−1.94, 0.07)

CI, confidence interval; DASS, Depression and Anxiety Stress Scale; DERS, Difficulties in Emotion Regulation Scale; PCL-5, PTSD Checklist for DSM-5.

About 50% of the participants (*n* = 33) were ready after the Trauma PORTAL to progress to a psychotherapy group in the TTP program. About 32% (*n* = 21) elected to repeat the content of the PORTAL intervention in a synchronous group. About 18% (*n* = 12) did not feel the need to continue with any additional intervention in the TTP. Eleven were lost to follow-up and one chose not to continue in the TTP after the study. Attending both components (i.e., attending the weekly groups and engaging in modules) was most beneficial in moving to the next steps in the TTP and was associated with a low discharge rate.

## Discussion

The Trauma PORTAL is a blended e-health intervention for adults designed to support a stepped approach to treating mental health sequelae of CIT. In this initial open-label feasibility study, recruitment was rapid, with 75.9% of potentially eligible participants enrolling, and there was a reasonable follow-up rate for post-intervention questionnaires. The intervention was well-received, with participants reporting that it provided valuable information and offered a positive and supportive experience overall. They highly ranked both the psychoeducational content and the multimedia format. Further, symptomatology improved at a clinically significant level from pre- to post-intervention.

The Trauma PORTAL, featuring asynchronous multimedia modules, was appreciated for its user-friendliness, with 95.9% of participants valuing the convenience of self-paced access. Notably, 98% reported that the intervention increased their access to health care. Engagement was high, with 87.9% engaging with either the online modules, weekly groups, or both, and 4 (6%) of the participants dropping out during the study. Our results align with the growing trend of patients embracing virtual interventions for their health care needs and research showing that blended interventions can enhance engagement.^40,41^ These findings are promising, especially given the historically high drop-out rates in trauma-focused therapies, particularly in outpatient clinic settings.^42–46^

Given the limited research on blended interventions incorporating a significant asynchronous component for adult survivors of CIT, the acceptability and safety of such an approach is still emerging.^47^ Our participants had a mean score of 5.03 (SD 2.33) on the ACE scale, indicating a significant prevalence of childhood trauma experiences. Importantly, our study revealed no safety concerns on the weekly feedback forms, and no participant withdrew for safety-related reasons. Additionally, 73% felt more prepared to engage in further trauma therapy after participating in the intervention. This promising finding underscores the safety and acceptability of incorporating a substantial asynchronous component within the intervention.

Our study found a high prevalence of not only PTSD but also depression and other disorders among participants, consistent with existing literature on comorbidity in individuals with CIT.^48,49^ Our secondary analysis revealed a significant decrease in PTSD symptoms and lower levels of depression and stress. Our results are similar to those reported in the literature that demonstrate that stabilization-focused interventions result in decreased depressive and complex PTSD symptoms and an increase in post-traumatic growth, which includes more self-reliance, confidence to handle difficulties, and an openness to new possibilities,^50–52^ though more research is needed.^53^ Since this study demonstrated feasibility, the next step will be a future RCT to help establish the effectiveness of the Trauma PORTAL intervention.

This pilot study also revealed some room for improvements in the protocol to be used for a future trial. In a future study, efforts to improve the diversity in the sample would be important, given that 60% of the participants identified as women, 92.4% as white, and 72.7% had a post-secondary education. The gender distribution in our study does align with the patient population of our program, which operates within an academic hospital with a particular emphasis on women’s health. In addition, we would like to use the Clinician-Administered PTSD Scale for DSM-5, in addition to the PCL-5, in a future study, as there is some discrepancy in the literature between the two measures, though they assess the same construct.^54^

## Conclusion

This study demonstrates the feasibility, usability, and acceptability of the Trauma PORTAL, a blended e-health intervention formulated as part of a stepped approach to care in trauma therapy. As e-health interventions continue to become accepted by patient populations, they may play an integral role in a stepped care model for psychotherapy delivery for many patients, some of whom may gain sufficient symptom remission with this step.^30^ Importantly, developing e-health interventions that can narrow the gap between wait time and treatment is crucial for improving access to care and health outcomes. The results support future investigations into the intervention’s clinical efficacy for individuals seeking trauma therapy. A future RCT of the Trauma PORTAL will compare the clinical outcomes of an intervention group with a treatment-as-usual control group, which will aid in further understanding the role of this blended e-health intervention in a scalable, stepped approach to care for this patient population.

## Data Availability

Data available from the corresponding author on request.

## Abbreviations Used

ACE: Adverse Childhood Experiences
CIT: Childhood Interpersonal Trauma
CIs: confidence intervals
DASS-21: Depression and Anxiety Stress Scale
DERS-18: Difficulties in Emotion Regulation Scale
MDs: mean differences
MINI: Mini International Neuropsychiatric Interview
PCL-5: PTSD Checklist for DSM-5
PORTAL: Providing Online Trauma Therapy using an Asynchronous Learning Platform
PTSD: Post-traumatic Stress Disorder
RCT: randomized controlled trial
REDCap: Research Electronic Data Capture
SD: standard deviation
TTP: Trauma Therapy Program
WCH: Women’s College Hospital
