# Supplementary material for: The Trauma PORTAL—A Blended e-Health Intervention for Survivors of Childhood Interpersonal Trauma: An Open-Label Pilot Study
Source: Telemed Rep. 2024 Jul 12;5(1):195–204. doi: 10.1089/tmr.2024.0020 (PMC11286000; doi:10.1089/tmr.2024.0020)
Supplement: Supplementary Appendix S1 [file tmr.2024.0020_ross_supplementaryappendix_sa1.docx]

Supplementary Material

*Supplementary Appendix SA1: Time spent on each of the 8 modules according to the weekly Iterative Feedback form*

| **Module** | **Time Spent on Module** | | | | | **Number of respondents** |
| --- | --- | --- | --- | --- | --- | --- |
|  | **0 mins** | **<30 mins** | **30-60 mins** | **1-2 hours** | **>2 hours** |  |
| Week 1 | 6.9% | 13.8% | 41.4% | 27.6% | 10.3% | 29 |
| Week 2 | 0.0% | 8.3% | 41.7% | 33.3% | 16.7% | 24 |
| Week 3 | 4.3% | 8.7% | 30.4% | 30.4% | 26.1% | 23 |
| Week 4 | 0.0% | 9.5% | 47.6% | 14.3% | 28.6% | 21 |
| Week 5 | 6.3% | 6.3% | 37.5% | 25.0% | 25.0% | 16 |
| Week 6 | 7.1% | 0.0% | 50.0% | 21.4% | 21.4% | 14 |
| Week 7 | 6.7% | 6.7% | 60.0% | 20.0% | 6.7% | 15 |
| Week 8 | 6.7% | 6.7% | 46.7% | 33.3% | 6.7% | 15 |
