# Supplementary material for: The Trauma PORTAL—A Blended e-Health Intervention for Survivors of Childhood Interpersonal Trauma: An Open-Label Pilot Study
Source: Telemed Rep. 2024 Jul 12;5(1):195–204. doi: 10.1089/tmr.2024.0020 (PMC11286000; doi:10.1089/tmr.2024.0020)
Supplement: Supplementary Appendix S2 [file tmr.2024.0020_ross_supplementaryappendix_sa2.docx]

*Supplementary Appendix SA2: General Internet Attitude Scale*

71.8% agreed that the Internet positively influences society; 17.2% had no opinion; the remaining 10.8% disagreed. 42.1% disagreed that using the Internet brings about feelings of exhilaration; 31.3% had no opinion on this, while 25.3% agreed. 52% agreed that there are negative effects, both at an individual and societal level, to using the Internet; 22.7% had no opinion, and 25.3% disagreed. 43.6% disagreed that they felt ‘bad’ when using the Internet; 25.8% had no opinion, and 30.3% agreed.
