# Supplementary figures and images for: The Trauma PORTAL—A Blended e-Health Intervention for Survivors of Childhood Interpersonal Trauma: An Open-Label Pilot Study
Source: Telemed Rep. 2024 Jul 12;5(1):195–204. doi: 10.1089/tmr.2024.0020 (PMC11286000; doi:10.1089/tmr.2024.0020)

*Supplementary Appendix SA3: Trauma PORTAL Module Content*


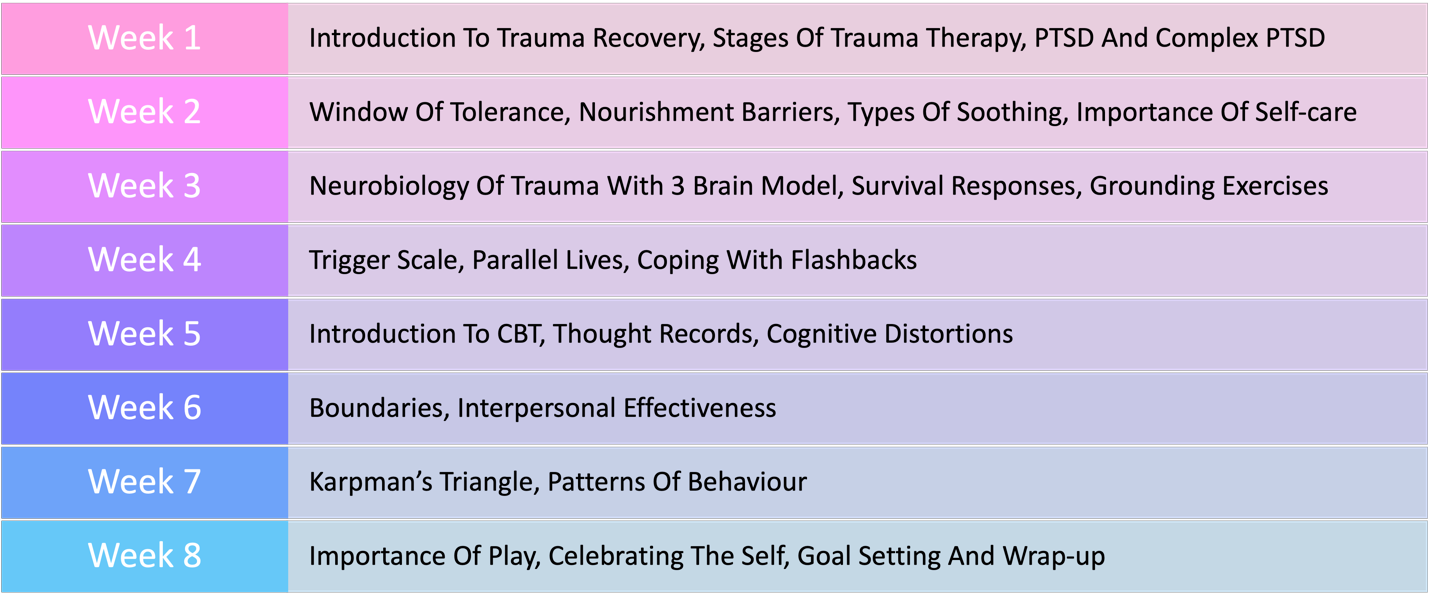

Supplement: Supplementary Appendix S3 [file tmr.2024.0020_ross_supplementaryappendix_sa3.docx]
